# Parasitological correlates of *Plasmodium ovale curtisi* and *Plasmodium ovale wallikeri* infection

**DOI:** 10.1186/s12936-016-1601-2

**Published:** 2016-11-10

**Authors:** Melissa S. Phuong, Rachel Lau, Filip Ralevski, Andrea K. Boggild

**Affiliations:** 1 McMaster University, Hamilton, Canada; 2 Public Health Ontario Laboratories, Toronto, Canada; 3 Tropical Disease Unit, Division of Infectious Diseases, Toronto General Hospital, University Health Network, 200 Elizabeth Street, 13EN-218, Toronto, ON M5G 2C4 Canada; 4 University of Toronto, Toronto, Canada

**Keywords:** *Plasmodium ovale*, Malaria diagnosis, Microscopy, PCR, Rapid antigen detection test

## Abstract

**Background:**

Malaria, due to *Plasmodium ovale*, can be challenging to diagnose due to clinically mild disease and low parasite burden. Two genetically distinct sub-species of *P. ovale* exist: *Plasmodium ovale curtisi* (classic) and *Plasmodium ovale wallikeri* (variant). It is presently unknown if the sub-species causing infection affects performance of malaria diagnostic tests. The aim of this work was to understand how the genetically distinct sub-species, *P. o. curtisi* and *P. o. wallikeri*, affect malaria diagnostic tests.

**Methods:**

*Plasmodium ovale*-positive whole blood specimens were sub-speciated by PCR and sequencing of 18S rRNA and *dhfr*-*ts*. Parasitaemia, morphology, pan-aldolase positivity, 18S copy number, and *dhfr*-*ts* sequences were compared between sub-species.

**Results:**

From 2006 to 2015, 49 *P. ovale* isolates were identified, of which 22 were *P. o. curtisi* and 27 *P. o. wallikeri*; 80% were identified in the last five years, and 88% were acquired in West Africa. Sub-species did not differ by parasitaemia, 18S copy number, or pan-aldolase positivity. Lack of Schüffner’s stippling was over-represented among *P. o. wallikeri* isolates (p = 0.02). Several nucleotide polymorphisms between the sub-species were observed, but they do not occur at sites believed to relate to antifolate binding.

**Conclusions:**

*Plasmodium ovale* is increasing among travellers to West Africa, although sub-species do not differ significantly by parasitologic features such as parasitaemia. Absence of Schüffner’s stippling may be a feature specific to *P. o. wallikeri* and is a novel finding.

## Background

*Plasmodium ovale* is one species of malaria parasites that is pathogenic to humans [1]. Prevalent in West and East Africa and the Asia–Pacific [1], *P. ovale* infections can be challenging to diagnose in part due to the typical presentation of clinically mild disease and low parasite burden [2]. Consequently, conventional diagnostic tools, such as microscopy of blood films and rapid diagnostic tests (RDT), have limited performance in detecting *P. ovale* infection [3–5], often resulting in the use of molecular techniques to confirm the diagnosis [5].

Two genetically distinct sub-species of *P. ovale* exist: *Plasmodium ovale curtisi* and *Plasmodium ovale wallikeri* [7, 8], which are known as the classic and variant types, respectively. First recognized in 2010, these two sub-species are believed to have diverged one to two million years ago [7, 9]. Clinical differences between the two sub-species have not been clearly elucidated, although there have been differences found in latency period [9] and thrombocytopaenia [10]. At present, it is unknown if the performance of malaria diagnostic tests may differ between the sub-species, causing infection. It is known that the dihydrofolate reductase-thymidylate synthase (*dhfr*-*ts*) domain, a common anti-malarial drug target and a region that can be linked to antifolate resistance, has been thoroughly investigated in *Plasmodium falciparum* and *Plasmodium vivax*. However, investigations into this region have only recently begun for *P. ovale* isolates [2, 8, 11]. Previous reports have implicated specific sites in the peptide sequence that may interfere with antifolate binding at the active site, which include D53, F57, S58, T61, S113, S116, I169, and T190 [8, 11]. R499/503 and C519/523 (*P. ovale curtisi*/*P. ovale wallikeri*) are also believed to be significant for thymidylate synthase activity, specifically [8]. As atovaquone-proguanil, the proguanil component of which is an antifolate, is one of the most commonly and empirically used anti-malarials for travel-acquired malaria, it is important to understand if mutations in regions of *dhfr*-*ts* known to confer resistance in other species of malaria are present in *P. ovale* isolates, and if these mutations are specific to one sub-species over another.

Further understanding of parasitologic correlates of *P. ovale* sub-species may have important implications for diagnosis and management. The potential differences in parasite burden, RDT performance, morphology, and molecular diagnosis between *P. ovale curtisi* and *P. ovale wallikeri* were investigated. The DHFR-TS peptide sequence was also compared between isolates to investigate potential mutations in regions known to confer antifolate resistance in other species of malaria.

## Methods

### Specimens

*Plasmodium ovale*-positive, whole blood specimens stored in the malaria biobank at Public Health Ontario Laboratories (PHOL) from October 2006 to July 2015 were identified and retrieved. For the staining procedure, 2 mL of standard Giemsa stain was diluted in 48 mL of phosphate buffer (pH 7.1) prior to use. The phosphate buffer consists of 65 mL of Na_2_HPO_4_, 35 mL of NaH_2_PO_4_, and 900 mL of distilled water, resulting in 4.35 and 2.33 mM concentrations, respectively. For thin films, the slide was immersed in methanol for 10 s, allowed to dry, and then immersed in the working Giemsa solution for 10 min. The slide was then immersed in phosphate buffer solution for 10 s and air dried. Thick films were immersed in the working Giemsa solution for 10 min, immersed in the phosphate buffer for 30 s, and air dried. With each working solution prepared, a control *P. falciparum* slide was prepared and examined. Parasitaemia, morphological features, pan-aldolase antigen-positivity as determined by the BinaxNOW Malaria test (Alere, Ottawa, ON, Canada), year of import, and country of acquisition that were recorded in the biobank database following initial diagnostic processing were collected and analysed. The study was approved by the Research Ethics Board of Public Health Ontario.

### DNA extraction

DNA of all specimens was extracted using the DNA Mini Kit Blood or Body Fluid Spin Protocol (Qiagen, Germantown, MD, USA). Prior to use, DNA was stored at −20 °C.

### Qualitative and quantitative real-time PCR

*Plasmodium falciparum*/*P. vivax* species-specific duplex [6] and *Plasmodium malariae*/*P. ovale* species-specific duplex real-time PCR (qPCR) assays were conducted to confirm microscopy species identification as previously described [12]. All qPCR assays were run under the following conditions using the ABI 7900HT qPCR system: 50 °C for 2 min, 95 °C for 10 min, 95 °C for 15 s, and 60 °C for 1 min (45 cycles); 12.5 µl of TaqMan universal PCR master mix (Life Technologies, Burlington, ON, Canada) and 5 µl of DNA primers and probes with concentrations as previously reported [6, 12, 13] were used, for a final volume of 25 µl per reaction. All qPCR amplification curves were analysed using a manual threshold cycle of 0.02 and an automatic baseline. A cycle threshold (Ct) of <38 was considered to be a positive result.

18S rRNA gene copy numbers were quantified by running the *P. malariae*/*P. ovale* species-specific duplex qPCR assay and including serial dilutions of a *P. ovale* clone (ATCC, Manassas, VA, USA) alongside specimen DNA in triplicates. A linear regression was then constructed based on the logarithm of the gene copy number and Ct values for each concentration of the clone. This equation could then be used to calculate the gene copy number for each banked specimen.

### Sequencing of 18S rRNA and dihydrofolate reductase-thymidylate synthase

Endpoint PCR of target regions, visualization of the amplicons on agarose gel, and Sanger sequencing were conducted as previously described [12]. Specifically, endpoint PCR of a 396-bp product from the 18S rRNA region was conducted with high-fidelity polymerase AccuPrime Pfx Supermix (Life Technologies, Burlington, ON, Canada) and 200 nM (each) of the primers Plasmo 18S forward (5′-ATTCAGATGTCAGAGGTGAAATTCT-3′) and Plasmo 18S reverse (5′-TCAATCCTACTCTTGTCTTAAACTA-3′). Using an ABI Veriti fast thermal cycler, the cycling conditions were 95 °C for 5 min, followed by 95 °C for 15 s, 58 °C for 30 s, and 68 °C for 30 s for 45 cycles, and then 68 °C for 5 min. Amplicons were visualized on 1% agarose gels with ethidium bromide prior to Sanger sequencing. For sequencing, the same forward and reverse primers were used along with a BigDye Terminator v3.1 cycle sequencing kit (Life Technologies, Burlington, ON, Canada), which was run according to the manufacturer’s recommended conditions. The products were purified by a BigDye XTerminator (Life Technologies, Burlington, ON, Canada) and analysed with an ABI 3130xl genetic analyzer. Forward and reverse sequences of each sample were aligned using Vector NTI software (Life Technologies, Burlington, ON, Canada), and the sequences were verified using a BLAST search to confirm the sub-species identification [14].

Primers for sequencing the *dhfr*-*ts* gene were newly designed for this study. All primers were designed using Primer3 [15, 16] and predominantly conserved regions of the complete sequence of the *P. ovale dhfr*-*ts* gene (GenBank: EU266602). Degenerate nucleotides were included based on the *dhfr*-ts gene for *P. o. curtisi* (GenBank: KP050414) and *P. o. wallikeri* (GenBank: KP050415). Endpoint PCR of the dihydrofolate reductase-thymidylate synthase region was conducted on all specimens with high-fidelity polymerase Phusion (New England BioLabs, Ipswich, MA, USA) and 200 nM (each) of the primers PocPowDHFR-exF (5′-YTCWACCTTCAGGGGTATCG-3′) and PocPowDHFR-exR (5′-AGTTTWAGCGTGGGRAAAGG-3′), generating an approximately 1700-bp product that covers the 99th amino acid to 1814–1826th amino acid of the sequence, where the end target varies based on the sub-species. The cycling conditions were 98 °C for 30 min, followed by 98 °C for 10 s, 64 °C for 30 s, and 72 °C for 1 min for 40 cycles, and then 72 °C for 10 min using an ABI Veriti fast thermal cycler. Visualization and Sanger sequencing of the PCR products were performed identically compared to the 18S rRNA methodology with the exception of the primers used. For sequencing, PocPowDHFR-exF, PocPowDHFR-exR, and a third internal primer, PocPowDHFR-inR (5′-TTTCCATTKGTTTCCCCTCT-3′) were used. Sequences for each specimen were aligned and verified using a BLAST search to further confirm the sub-species identification. Alignments of *P. ovale* sequences were performed using Mega6 software [17]. Genetic and peptide sequences between the enrolled specimens and complete *P. ovale* sequences that were previously reported (GenBank: EU266601, EU266604–EU266618, GQ250090, GQ250091, KP050409, and KP05413–KP050415) [2, 7, 18] were compared.

### Statistical analysis

Reported parasitaemia, morphological features (including Schüffner’s stippling), pan-aldolase antigen-positivity, 18S gene copy number, year of import, and region of acquisition were compared between the two sub-species. Parasitaemia was treated as categorical data by recording if the percentage of parasites observed were <0.1 or ≥0.1%, as the former category is a convention used by PHOL laboratory technicians. Categorical variables were compared using Yates’ corrected Chi squared analysis or Fisher’s exact test, and continuous variables using Mann–Whitney U tests using GraphPad Prism 6 (GraphPad Software, CA, USA). Level of significance was set at p < 0.05.

## Results

Of 49 isolates of *P. ovale* identified from October 2006 to July 2015, ten (20.4%) were identified through 2010, while 39 (79.6%) were identified in or after the year 2011, with 24 (49%) identified in 2013 or 2014, the last two full years in the enrolment period. Country of exposure data were available for 17/49 (34.7%) specimens, with Nigeria being the most well represented source country (n = 9, 18.4%). Of the 17 isolates with an attached travel history, 15 (88.2%) were acquired in West Africa, with represented source countries of Nigeria, Ghana (n = 2), Ivory Coast (n = 1), Liberia (n = 1), Sierra Leone (n = 1), Congo (n = 1), and Cameroon (n = 1). Two isolates were imported from East or Saharan Africa, one each from Tanzania and the Sudan.

**Fig. 1:**
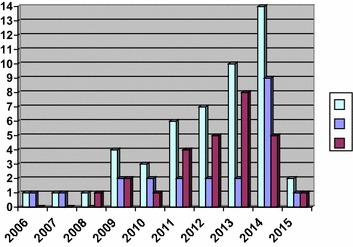
*Plasmodium ovale* isolates confirmed by Public Health Ontario Laboratories by year of import. 2006 and 2015 represent partial calendar years (October–December 2006, and January–June 2015). Emergence of *P. ovale* was equally distributed among the two sub-species, with six (27.3%) isolates of *P. ovale curtisi* identified prior to 2011, and 16 (72.7%) in or after 2011, compared to four (14.8%) isolates of *P. ovale wallikeri* identified prior to 2011, and 23 (85.2%) in or after 2011 (p = 0.31)

Of 49 total isolates, 22 *P. ovale curtisi* and 27 *P. ovale wallikeri* isolates were identified by 18S rRNA sequencing (Table 1). Region of acquisition did not differ between sub-species (p = 1.00). Both sub-species appeared to emerge simultaneously in the past five years, with no difference between sub-species: six (27.3%) isolates of *P. ovale curtisi* were identified prior to 2011, and 16 (72.7%) in or after 2011, compared to four (14.8%) isolates of *P. ovale wallikeri* identified prior to 2011, and 23 (85.2%) in or after 2011 (p = 0.31) (Fig. 1).

**Table 1:** Pan-aldolase antigen-positivity (T2), parasitaemia, 18S rRNA gene copy number, reported Schüffner’s stippling, and the number of days between blood collection and processing *Plasmodium ovale curtisi* and *Plasmodium ovale wallikeri* specimens are recorded

Specimen number	Sub-species	Aldolase	Parasitaemia (%)	18S rRNA gene copy number/µL blood	Schüffner’s stippling	Number of days between blood collection and processing	Year of import	Country of acquisition
1	*P. ovale curtisi*	+	0.8	11,649	+	1	2014	Ivory Coast
2	*P. ovale curtisi*	+	0.6	5054	+	1	2014	Nigeria
3	*P. ovale curtisi*	−	0.3	2132	+	7	2014	Unknown
4	*P. ovale curtisi*	+	0.3	1928	+	1	2014	Unknown
5	*P. ovale curtisi*	+	0.2	16,807	+	0	2012	Unknown
6	*P. ovale curtisi*	+	0.2	626	+	0	2014	Unknown
7	*P. ovale curtisi*	+	0.2	831	+	0	2014	Unknown
8	*P. ovale curtisi*	+	0.1	3093	NA	NA	2006	Unknown
9	*P. ovale curtisi*	+	0.1	8445	+	1	2011	Ghana; Nigeria
10	*P. ovale curtisi*	−	<0.1	8264	+	1	2009	Unknown
11	*P. ovale curtisi*	−	<0.1	3285	+	NA	2009	Nigeria
12	*P. ovale curtisi*	−	<0.1	1765	+	0	2010	Unknown
13	*P. ovale curtisi*	−	<0.1	931	+	0	2010	Unknown
14	*P. ovale curtisi*	−	<0.1	1972	+	0	2011	Ghana
15	*P. ovale curtisi*	+	<0.1	4962	+	NA	2007	Unknown
16	*P. ovale curtisi*	−	<0.1	302	+	1	2012	Unknown
17	*P. ovale curtisi*	−	<0.1	855	+	0	2014	Nigeria
18	*P. ovale curtisi*	+	<0.1	2088	+	1	2015	Nigeria
19	*P. ovale curtisi*	−	<0.1	538	+	1	2013	Tanzania
20	*P. ovale curtisi*	+	<0.1	2991	+	NA	2014	Unknown
21	*P. ovale curtisi*	−	<0.1	325	+	1	2014	Unknown
22	*P. ovale curtisi*	−	<0.1	2637	+	1	2013	Unknown
23	*P. ovale wallikeri*	−	0.5	12,709	−	0	2011	Unknown
24	*P. ovale wallikeri*	+	0.5	2835	−	0	2014	Unknown
25	*P. ovale wallikeri*	+	0.4	5013	+	0	2013	Unknown
26	*P. ovale wallikeri*	+	0.3	7107	+	1	2008	Unknown
27	*P. ovale wallikeri*	−	0.3	6826	+	0	2011	Sudan
28	*P. ovale wallikeri*	+	0.3	15,840	+	4	2012	Liberia
29	*P. ovale wallikeri*	+	0.3	8697	+	0	2012	Nigeria
30	*P. ovale wallikeri*	+	0.3	3897	+	0	2014	Unknown
31	*P. ovale wallikeri*	−	0.15	114	+	1	2013	Unknown
32	*P. ovale wallikeri*	+	0.1	2160	+	0	2013	Unknown
33	*P. ovale wallikeri*	−	0.1	91	+	0	2014	Unknown
34	*P. ovale wallikeri*	−	<0.1	985	−	0	2009	Unknown
35	*P. ovale wallikeri*	+	<0.1	4402	+	1	2010	Unknown
36	*P. ovale wallikeri*	−	<0.1	752	+	1	2011	Unknown
37	*P. ovale wallikeri*	−	<0.1	935	+	0	2011	Sierra Leone
38	*P. ovale wallikeri*	−	<0.1	538	+	1	2012	Unknown
39	*P. ovale wallikeri*	−	<0.1	739	−	1	2013	Unknown
40	*P. ovale wallikeri*	NA	<0.1	4	+	2	2009	Nigeria
41	*P. ovale wallikeri*	−	<0.1	364	+	0	2012	Congo
42	*P. ovale wallikeri*	+	<0.1	1898	−	NA	2013	Cameroon
43	*P. ovale wallikeri*	+	<0.1	435	+	0	2014	Unknown
44	*P. ovale wallikeri*	−	<0.1	51	+	0	2013	Nigeria
45	*P. ovale wallikeri*	+	<0.1	45	+	1	2014	Unknown
46	*P. ovale wallikeri*	−	<0.1	256	−	1	2013	Nigeria
47	*P. ovale wallikeri*	−	<0.1	488	+	0	2013	Unknown
48	*P. ovale wallikeri*	−	<0.1	187	−	NA	2012	Unknown
49	*P. ovale wallikeri*	−	<0.1	571	−	0	2015	Unknown

*NA* not available

### Microscopy and RDT

Records of pan-aldolase antigen result and morphological features were absent for one specimen, resulting in the omission of this isolate from relevant calculations. Median parasitaemia (by microscopy) of specimens positive for *P. ovale curtisi* was <0.1% (range <0.1–0.8%), while for *P. ovale wallikeri* it was <0.1% (range <0.1–0.5%), and no difference was found (p = 0.99). There was no difference found in pan-aldolase antigen-positivity, where it was detectable in 11 (50.0%) specimens containing *P. ovale curtisi* and 11 (42.3%) specimens containing *P. ovale wallikeri* (p = 0.81). Pan-aldolase positivity for all specimens was 45.8%.

**Fig. 2:**
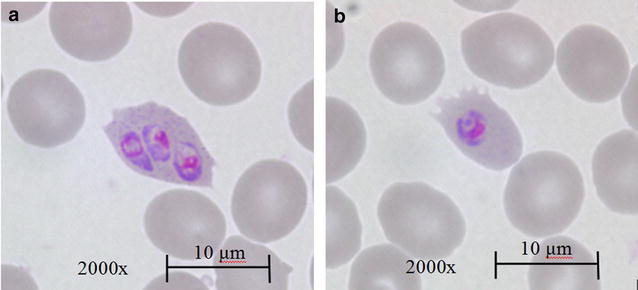
Giemsa-stained microscopy of *Plasmodium ovale.* Note prominent Schüffner’s stippling in **a**, compared to the absence of Schüffner’s stippling in **b**

When comparing morphological features (Table 2), it was noted that all eight *P. ovale* parasites without Schüffner’s stippling were *P. ovale wallikeri* (p = 0.02) (Fig. 2). Parasitaemia (p = 0.20), 18S rRNA gene copy number (p = 0.50), and pan-aldolase antigen positivity (p = 0.40), were not found to be differentially associated with Schüffner’s stippling. Data on time between specimen collection and receipt were missing for two *P. ovale wallikeri* specimens without Schüffner’s stippling, and four *P. ovale curtisi* specimens, where three *P. ovale curtisi* specimens had Schüffner’s stippling and the other specimen had no morphological features recorded. No significant difference in time for specimen processing between specimens with and without Schüffner’s stippling was noted (p = 0.57).

**Table 2:** Number of isolates with Schüffner’s stippling noted at Giemsa-stained microscopy of specimens positive for *Plasmodium ovale*

*Plasmodium ovale* sub-species	Schüffner’s stippling noted	Schüffner’s stippling not noted
*Plasmodium ovale curtisi*	21	0
*Plasmodium ovale wallikeri*	19	8

## 18S gene copy number

18S rRNA gene copy number was found to be not normally distributed among all samples based on the D’Agostino-Pearson normality test, p < 0.0001. Median 18S rRNA gene copy number for *P. ovale curtisi* and *P. ovale wallikeri* were 2110/µL (range 302–16,807/µL) and 844/µL (range 4–15,840/µL), respectively (p = 0.09).

### Dihydrofolate reductase-thymidylate synthase (*dhfr*-*ts*)

Partial sequences of *dfhr*-*ts* could be obtained in 16 *P. ovale wallikeri* and seven *P. ovale curtisi*. In 26/49 isolates, the *dhfr*-*ts* region selected could not be amplified by the selected primer set. Several differences in the peptide sequences between *P. ovale curtisi* and *P. ovale wallikeri* were observed (Table 3). Non-synonymous mutations were observed within the *P. ovale wallikeri* isolates as previously reported [2]. Specifically, deletions of two or four amino acids (i.e., TA252–253 or TATA252–255) were identified in six and one *P. ovale wallikeri* isolates, respectively (Table 3). Furthermore, due to an ambiguity in the nucleic acid sequence, potential Y359F and Y366F mutations were observed. Finally, for one specimen, a H285Q mutation was recorded for one *P. ovale wallikeri* isolate. While Y359F and Y366F mutations are not expected to change any amino acid properties, a H285Q indicates a change from a basic histidine to a polar, uncharged glutamine. Among the sequences analysed in this study, polymorphisms specific to either sub-species were not observed (Table 4). One *P. ovale wallikeri* specimen did have a I169L mutation, although this does not reflect any changes in amino acid properties.

**Table 3:** Discrepant amino acids at the *Plasmodium ovale* DHFR-TS domain, either between sub-species or within sub-species

Specimen number	Amino acid number in Dhfr-ts domain																									
	187	204	207	209	252–255	267	269	280	285	296	300	306	309	320	339	342	350	355	359	366	370	374	409	450	482	501
2	V	E	K	T	DELETED	D	T	Y	H	V	S	K	T	E	G	A	S/T	P/Q	Y	Y	I	N/K	ND	ND	ND	ND
4	V	E	K	T	DELETED	D	T	Y	H	V	S	K	T	E	G	A	T	Q	Y	Y	I	K	ND	ND	ND	ND
8	V	E	K	T	DELETED	D	T	Y	H	V	S	K	T	E	G	A	T	Q	Y	Y	I	K	I	N	D	L
9	V	E	K	T	DELETED	D	T	Y	H	V	S	K	T	E	G	A	T	Q	Y	Y	I	K	I	N	ND	ND
17	ND	ND	ND	ND	ND	ND	ND	ND	ND	V	S	K	T	E	G	A	T	Q	Y	Y	I	K	ND	ND	ND	ND
21	V	E	K	T	DELETED	D	T	Y	H	V	S	K	T	E	G	A	T	Q	Y	Y	I	K	ND	ND	ND	ND
22	V	E	K	T	DELETED	D	T	Y	H	V	S	K	T	E	G	A	T	Q	Y	Y	I	K	I	N	D	L
*P. ovale curtisi* KP050414.1	V	E	K	T	DELETED	D	T	Y	H	V	S	K	T	E	G	A	T	Q	Y	Y	I	K	I	N	D	L
*P. ovale curtisi* KP050413.1	V	E	K	T	DELETED	D	T	Y	H	V	S	K	T	E	G	A	T	Q	Y	Y	I	K	I	N	D	L
*P. ovale curtisi* EU266611	V	E	K	T	DELETED	D	T	Y	H	V	S	K	T	E	G	A	T	Q	Y	Y	I	K	I	N	D	L
*P. ovale curtisi* EU266608	V	E	K	T	DELETED	D	T	Y	H	V	S	K	T	E	G	A	T	Q	Y	Y	I	K	I	N	D	L
*P. ovale curtisi* EU266609	V	E	K	T	DELETED	D	T	Y	H	V	S	K	T	E	G	A	T	Q	Y	Y	I	K	I	N	D	L
*P. ovale curtisi* EU266610	V	G	K	T	DELETED	D	T	Y	H	V	S	K	T	E	G	A	T	Q	Y	Y	I	K	I	N	D	L
*P. ovale curtisi* EU266606	V	E	K	T	DELETED	D	T	Y	H	V	S	K	T	E	G	A	T	Q	Y	Y	I	K	I	K	D	L
*P. ovale curtisi* EU266607	V	E	K	T	DELETED	D	T	Y	H	V	S	K	T	E	G	A	T	Q	Y	Y	I	K	I	N	D	L
23	I	D	E	A	TATA	A	S	F	Q	G	G	G	A	D	M	V	S	Q	Y	Y	M	K	V	K	E	T
24	I	D	E	A	TATA	A	S	F	H	G	G	G	A	D	M	V	S	Q	Y	Y	M	K	V	K	E	T
26	I	D	E	A	TATA	A	S	F	H	G	G	G	A	D	M	V	S	Q	Y/F	Y/F	M	K	V	K	E	T
27	I	D	E	A	DELETED	A	S	F	H	G	G	G	A	D	M	V	S	Q	Y/F	Y/F	M	K	V	K	E	T
28	I	D	E	A	TATA	A	S	F	H	G	G	G	A	D	M	V	S	Q	Y	Y	M	K	V	K	E	T
29	I	D	E	A	TA–	A	S	F	H	G	G	G	A	D	M	V	S	Q	Y	Y	M	K	V	K	E	T
30	I	D	E	A	TATA	A	S	F	H	G	G	G	A	D	M	V	S	Q	Y	Y	M	K	V	K	E	T
33	I	D	E	A	TA–	A	S	F	H	G	G	G	A	D	M	V	S	Q	Y/F	Y	M	K	V	K	E	T
34	I	D	E	A	TATA	A	S	F	H	G	G	G	A	D	M	V	S	Q	Y	Y	M	K	V	K	E	T
35	I	D	E	A	TATA	A	S	F	H	G	G	G	A	D	M	V	S	Q	Y	Y	M	K	V	K	E	T
37	I	D	E	A	TATA	A	S	F	H	G	G	G	A	D	M	V	S	Q	Y	Y	M	K	V	K	E	T
39	I	D	E	A	TA–	A	S	F	H	G	G	G	A	D	M	V	S	Q	Y	Y	M	K	V	K	E	T
41	I	D	E	A	TA–	A	S	F	H	G	G	G	A	D	M	V	S	Q	Y	Y	M	K	V	K	E	T
42	I	D	E	A	TATA	A	S	F	H	G	G	G	A	D	M	V	S	Q	Y	Y	M	K	V	K	E	T
43	I	D	E	A	TA–	A	S	F	H	G	G	G	A	D	M	V	S	Q	Y	Y	M	K	V	K	E	T
44	I	D	E	A	TATA	A	S	F	H	G	G	G	A	D	M	V	S	Q	Y	Y	M	K	V	K	ND	ND
*P. ovale wallikeri* KP050415.1	I	D	E	A	TATA	A	S	F	H	G	G	G	A	D	M	V	S	Q	Y	Y	M	K	V	K	E	T
*P. ovale wallikeri* KP050409.1	I	D	E	A	TA–	A	S	F	H	G	G	G	A	D	M	V	S	Q	Y	Y	M	K	V	K	E	T
*P. ovale wallikeri* EU266612	I	D	E	A	TA–	A	S	F	H	G	G	G	A	D	M	V	S	Q	Y	Y	M	K	V	K	E	T
*P. ovale wallikeri* EU266613	I	D	E	A	TA–	A	S	F	H	G	G	G	A	D	M	V	S	Q	Y	Y	M	K	V	K	E	T
*P. ovale wallikeri* EU266614	I	D	E	A	TA–	A	S	F	H	G	G	G	A	D	M	V	S	Q	Y	Y	M	K	V	K	E	T
*P. ovale wallikeri* EU266615	I	D	E	A	TA–	A	S	F	H	G	G	G	A	D	M	V	S	Q	Y	Y	M	K	V	K	E	T
*P. ovale wallikeri* EU266617	I	D	E	T	TA–	A	S	F	H	G	G	G	A	D	M	V	S	Q	Y	Y	M	K	V	K	E	T
*P. ovale wallikeri* GQ250090	I	D	E	A	TA–	A	S	F	H	G	G	G	A	D	M	V	S	Q	Y	Y	M	K	V	K	E	T
*P. ovale wallikeri* EU266616	I	D	E	A	TA–	A	S	F	H	G	G	G	A	D	M	V	S	Q	Y	Y	M	K	V	K	E	T
*P. ovale wallikeri* EU266618	I	D	E	A	TA–	A	S	F	H	G	G	G	A	D	M	V	S	Q	Y	Y	M	K	V	K	E	T
*P. ovale wallikeri* GQ250091	I	D	E	A	TA–	A	S	F	H	G	G	G	A	D	M	V	S	Q	Y	Y	M	K	V	K	E	T
*P. ovale wallikeri* EU266601	I	D	E	A	TA–	A	S	F	H	G	G	G	A	D	M	V	S	Q	Y	Y	M	K	V	K	E	T
*P. ovale wallikeri* EU266604	I	D	E	A	TATA	A	S	F	H	G	G	G	A	D	M	V	S	Q	Y	Y	M	K	V	K	E	T
*P. ovale wallikeri* EU266603	I	D	E	A	TA–	A	S	F	H	G	G	G	A	D	M	V	S	Q	Y	Y	M	K	V	K	E	T
*P. ovale wallikeri* EU266605	I	D	E	A	TATA	A	S	F	H	G	G	G	A	D	M	V	S	Q	Y	Y	M	K	V	K	E	T

*ND* not determined

**Table 4:** Amino acids at the Dhfr-ts domain believed to affect antifolate binding

*Plasmodium ovale* isolate	Amino acid number in Dhfr-ts domain relevant to antifolate binding									
	53	57	58	61	113	116	169	190	503	523
*P. ovale curtisi* KP050414.1										
2	ND	ND	ND	ND	ND	ND	I	T	ND	ND
4	ND	F	S	T	S	S	I	T	R	C
8	ND	F	S	T	S	S	I	T	R	C
9	ND	F	S	T	S	S	I	T	R	C
17	ND	ND	ND	ND	ND	ND	ND	ND	ND	ND
21	ND	ND	ND	ND	ND	ND	I	T	ND	ND
22	ND	F	S	T	S	S	I	T	R	C
*P. ovale curtisi* EU266611	D	F	S	T	S	S	I	T	R	C
*P. ovale curtisi* EU266608	D	F	S	T	S	S	I	T	R	C
*P. ovale curtisi* EU266609	D	F	S	T	S	S	I	T	R	C
*P. ovale curtisi* EU266610	D	F	S	T	S	S	I	T	R	C
*P. ovale curtisi* EU266606	D	F	S	T	S	S	I	T	R	C
*P. ovale curtisi* EU266607	D	F	S	T	S	S	I	T	R	C
23	ND	ND	ND	ND	S	S	I	T	R	C
24	D	F	S	T	S	S	I	T	R	C
26	ND	F	S	T	S	S	L	T	R	C
27	ND	ND	ND	ND	S	S	I	T	R	C
28	ND	F	S	T	S	S	I	T	R	C
29	ND	ND	ND	ND	S	S	I	T	R	C
30	D	F	S	T	S	S	I	T	R	C
33	ND	F	S	T	S	S	I	T	R	C
34	ND	F	S	T	S	S	I	T	R	C
35	D	F	S	T	S	S	I	T	R	C
37	D	F	S	T	S	S	I	T	R	C
39	ND	F	S	T	S	S	I	T	R	C
41	D	F	S	T	S	S	I	T	R	C
42	ND	F	S	T	S	S	I	T	R	C
43	ND	ND	ND	ND	S	S	I	T	R	C
44	ND	ND	ND	ND	S	S	I	T	R	C
*P. ovale wallikeri* KP050415.1	D	F	S	T	S	S	I	T	R	C
*P. ovale wallikeri* KP050409.1	D	L	R	T	S	S	I	T	R	C
*P. ovale wallikeri* EU266612	D	F	S	T	S	S	I	T	R	C
*P. ovale wallikeri* EU266613	D	F	S	T	S	S	I	T	R	C
*P. ovale wallikeri* EU266614	D	F	S	T	S	S	I	T	R	C
*P. ovale wallikeri* EU266615	D	F	S	T	S	S	I	T	R	C
*P. ovale wallikeri* EU266617	D	F	S	T	S	S	I	T	R	C
*P. ovale wallikeri* GQ250090	D	F	S	T	S	S	I	T	R	C
*P. ovale wallikeri* EU266616	D	F	S	T	S	S	I	T	R	C
*P. ovale wallikeri* EU266618	D	F	S	T	S	S	I	T	R	C
*P. ovale wallikeri* GQ250091	D	F	S	T	S	S	I	T	R	C
*P. ovale wallikeri* EU266601	D	F	S	T	S	S	I	T	R	C
*P. ovale wallikeri* EU266604	D	F	S	T	S	S	I	T	R	C
*P. ovale wallikeri* EU266603	D	F	S	T	S	S	I	T	R	C
*P. ovale wallikeri* EU266605	D	F	S	T	S	S	I	T	R	C

*ND* not determined

## Discussion

*Plasmodium ovale* is increasingly observed among travellers, and due to its ability to relapse months to years after primary infection; unlike the more common and severe *P. falciparum*, its ability to inflict acute morbidity continues long after the immediate post-travel period. The data reported herein underscore the anecdotal impression among laboratory personnel that *P. ovale* is increasing among travellers returning from West Africa, with almost 80% of imports to PHOL occurring in the last five years. Between 2007 and 2012, *P. ovale* diagnoses climbed from one per year to eight per year at PHOL [12]. Although it appeared in this study that many more *P. ovale wallikeri* isolates were imported in the past five years compared to *P. ovale curtisi*, this difference was not significant, although with higher numbers over a longer enrolment period such a difference may emerge.

Due to widespread attrition of trained microscopists in laboratories of non-malaria endemic regions, such as North America, diagnosis of malaria is becoming increasingly reliant on RDT and molecular methods. However, given the extremely poor sensitivity of standard RDT for *P. ovale* infection [19], as well as typically low parasitaemia, under- and delayed diagnosis are increasingly likely. In addition, diagnostic morphologic features may not be demonstrable in the setting of extremely low parasitaemia, prompting empiric use of anti-malarials for a patient whose malaria screen is initially reported as “*Plasmodium* spp.” pending confirmatory molecular analysis. Standard choice of empiric oral therapy for uncomplicated malaria in North America is usually either atovaquone–proguanil (AP), or more rarely, artemether-lumefantrine. As resistance to the proguanil component of AP is conferred in *P. falciparum* by step-wise mutations of the *Dhfr* gene, understanding whether or not similar mutations can accumulate in *P. ovale*, and whether such mutations are over-represented among a particular sub-species of *P. ovale*, is important for informing empiric choice of anti-malarials in a patient with unspeciated malaria.

Among peptide sequences of DHFR-TS in isolates of *P. ovale curtisi* and *P. ovale wallikeri* studied herein, mutations known to confer antifolate resistance in other species of malaria, notably *P. falciparum* were not demonstrated Furthermore, non-synonymous mutations within each sub-species, *P. ovale wallikeri* in particular, were also observed, confirming previous reports [2]. While there are variations in the peptide sequences between sub-species, it is unknown how these polymorphisms influence enzyme activity. Amino acids in the DHFR-TS that are believed to be involved in antifolate binding, and which are believed to be synonymous with amino acids found in other *Plasmodium* species, were found to be consistent throughout all examined *P. ovale* specimens, regardless of sub-species. These findings suggest that the lack of clinical treatment failures observed in patients with *P. ovale* correlate well to gene sequences free of known markers of resistance, and are reassuring from the perspective of empiric choice of anti-malarials. It would be very unlikely, and unfortunate, for a traveller from West Africa with unspeciated malaria to be started empirically on chloroquine, which is actually the drug of choice for *P. ovale*, rather than AP.

Many reports have documented the existence of the two sub-species of *P. ovale*; it was previously unknown if parasitaemia, 18S rRNA gene copy number and the detection of the pan-aldolase antigen in BinaxNow RDT could differ between them. The results reported herein indicate that *P. ovale curtisi* and *P. ovale wallikeri* do not differ significantly by parasitaemia, pan-aldolase antigen-positivity or 18S rRNA gene copy number. A general low pan-aldolase antigen-positivity rate among all specimens was observed, which is consistent with previous literature [19, 20]. A lack of discernible Schüffner’s stippling may be a feature specific to *P. ovale wallikeri*, and may further challenge microscopists who rely on this as one of the features to distinguish *P. ovale* from the similarly low parasitaemic *P. malariae*, particularly when not all differentiating morphologies are present on the slide. As previously noted, *P. ovale wallikeri* cases appeared to increase more so than *P. ovale curtisi* in the past five years, and if this trend were to continue, the lack of Schüffner’s stippling may further hinder interpretation of microscopy and RDT results. The lack of Schüffner’s stippling among *P. ovale wallikeri* isolates joins other findings that the sub-species have distinguishing clinical characteristics, such as latency and degree of thrombocytopaenia, in addition to their genotype [9, 10]. That said, molecular techniques are still the most accurate and specific method for determining sub-species of *P. ovale*.

Limitations of the study include a relatively small sample size which limited the ability to comment on the possible emergence of variant over classic *P. ovale*, an inability to investigate DHFR-TS enzyme activity, and possible variation in Schüffner’s stippling visualization due to factors such as the age of the Giemsa stain or operator experience. As well, the attempt to account for time for specimen processing may be limited in terms of precision, as the exact time when blood was collected from patients was unavailable, meaning that this variable could only be analysed on the order of days rather than hours, the latter of which would have been ideal. Full clinical linkage of specimens was lacking and therefore data on latency of *P. ovale* infection or full travel details were unavailable. The limited availability of gene sequences in GenBank also restricted the ability to investigate the full *dhfr*-*ts* region and other potential virulence factors that could theoretically differ between the sub-species.

Future studies, including whole genome sequencing of *P. ovale*, will facilitate identification of genes of other known *Plasmodium* virulence factors that have not been previously investigated in *P. ovale*. In particular, nucleotide and peptide heterogeneity between the two sub-species can be further explored across a larger sample size to extrapolate potential consequences in protein structure. Clinical outcomes can also be investigated prospectively to determine whether specific polymorphisms have any effect on delayed parasite clearance and treatment failure. Future prospective investigation of the Schüffner’s stippling finding in *P. ovale wallikeri versus P. ovale curtisi* by diligent exclusion of competing factors, such as time to specimen processing, would further add to the literature around morphological identification, considering that microscopy remains the gold standard diagnostic test in malaria, and is often the only diagnostic test available in under-resourced settings.

## Conclusions

This study demonstrates that the two *P. ovale* sub-species, *P. ovale curtisi* and *P. ovale wallikeri*, are increasing among travellers to West Africa, and do not differ significantly by parasitaemia, RDT positivity, or 18S rRNA gene copy number, and may therefore be treated similarly from a clinical management perspective. A lack of discernible Schüffner’s stippling on microscopic examination may be a feature specific to *P. ovale wallikeri,* and one which may significantly challenge the diagnosis of this typically low parasitaemic species, especially as loss of technical expertise in diagnostic laboratories continues. Although nucleotide polymorphisms in *dhfr*-*ts* were identified, it is unknown if they could lead to antifolate resistance, as they did not occur in regions known to confer antifolate resistance in other species such as *P. falciparum*. Further interrogation of the relevance of such genetic differences is warranted.
